# The Birth Memories and Recall Questionnaire (BirthMARQ): development and evaluation

**DOI:** 10.1186/1471-2393-14-211

**Published:** 2014-06-20

**Authors:** Suzanne Foley, Rosalind Crawley, Stephanie Wilkie, Susan Ayers

**Affiliations:** 1 School of Psychology, University of Sussex, Sussex BN1 9QH, UK; 2 Department of Psychology, University of Sunderland, Sunderland SR6 0DD, UK; 3 Centre for Maternal and Child Health Research, City University London, London EC1R 1UW, UK

**Keywords:** Childbirth, Memory characteristics, Birth memories, Postnatal depression, Postnatal PTSD

## Abstract

**Background:**

Childbirth is a challenging and emotive experience that is accompanied by strong positive and/or negative emotions. Memories of birth may be associated with how women cognitively process birth events postpartum and potentially their adaptation to parenthood. Characteristics of memories for birth may also be associated with postnatal psychological wellbeing. This paper reports the development and evaluation of a questionnaire to measure characteristics of memories of childbirth and to examine the relationship between memories for birth and mental health.

**Methods:**

The Birth Memories and Recall Questionnaire (BirthMARQ) was developed by generating items from literature reviews and general measures of memory characteristics to cover dimensions relevant to childbirth. Fifty nine items were administered to 523 women in the first year after childbirth (*M* = 23.7 weeks) as part of an online study of childbirth. Validity of the final scale was checked by examining differences between women with and without probable depression and PTSD.

**Results:**

Principal components analysis identified 23 items representing six aspects of memory accounting for 64% of the variance. These were: *Emotional memory*, *Centrality of memory to identity, Coherence, Reliving, Involuntary recall*, and *Sensory memory*. Reliability was good (*M* alpha = .80). Women with probable depression or PTSD reported more emotional memory, centrality of memories and involuntary recall. Women with probable depression also reported more reliving, and those with probable PTSD reported less coherence and sensory memory.

**Conclusion:**

The results suggest the BirthMARQ is a coherent and valid measure of the characteristics of memory for childbirth which may be important in postnatal mood and psychopathology. While further testing of its reliability and validity is needed, it is a measure capable of becoming a valuable tool for examining memory characteristics in the important context of childbirth.

## Background

Childbirth is a significant and emotive life event for women and their partners [1]. For a proportion of parents, childbirth can be experienced as traumatic [2] and the perinatal period is associated with increased vulnerability to psychological symptoms, such as depression, anxiety and post-traumatic stress disorder (PTSD). It is estimated that up to 19% of women may suffer from postnatal depression [3], up to 16% from anxiety disorders [4], and up to 7% from PTSD after birth [2,5,6].

How women remember the events of birth is likely to be affected by a range of factors, including the intensity of the birth experience, how women appraise birth events, and postnatal mood or psychological disorders [7,8]. It is possible that characteristics of memories of birth may also be associated with the way women process the events of birth postpartum and their adaptation to parenthood. Research on memory for other autobiographical events shows memory characteristics are affected by the valence and intensity of emotion experienced at the time of the event [9-14] and by psychological symptoms, in particular PTSD and depression [12,15-20].

While these studies demonstrate the influence of emotion and psychological problems on memory characteristics, the evidence is not always consistent. This may be partly because these variables have been confounded with the type of event being remembered. The advantage of studying memories for childbirth is that this one event can vary in emotional valence and intensity, perceived trauma, and associated psychological symptoms. Thus, women’s memories of childbirth are not only interesting in their own right but also because they can inform debates about the association between memory characteristics and emotion, perceived trauma and psychological symptoms.

So far very few studies have specifically addressed the relationship between birth memory characteristics and postnatal psychological symptoms. One study examining memories of birth and postnatal PTSD found that self-reported memory disorganisation was associated with PTSD symptoms in women six weeks postpartum [7]. More negative birth experiences and less self-referent processing were associated with memory disorganisation. In addition, a qualitative study research by Ayers [8] found that women experiencing a traumatic birth reported fragmented memories of childbirth. Some evidence suggests that fragmented memory is associated with PTSD in other populations [16] but the evidence is not consistent. For example, Berntsen *et al.*[15] found no difference in rated fragmentation of memories for trauma in students with or without a PTSD profile.

A difficulty in examining memories for birth is that there is no measure developed specifically for use with childbirth. Questionnaires developed for general use, such as the Memory Characteristics Questionnaire [21] and the Autobiographical Memories Questionnaire [22], incorporate items measuring a variety of memory characteristics that are usually analysed as separate items [23]. More recently, Sutin and Robins [24] developed the Memory Experiences Questionnaire based on psychometric principles. However, general measures such as these are limited in application to childbirth because they were not developed or validated for use with postnatal women and omit items that might be important to childbirth memories and adjustment, such as self-referencing [7]. The current study therefore aimed to develop a questionnaire specifically for measuring the characteristics of memories of childbirth that may be important in postnatal mood and psychopathology.

## Methods

### Design

Items for the Birth Memories and Recall Questionnaire (BirthMARQ) were administered as part of a larger cross-sectional internet study of memories of birth and postnatal psychopathology. Self-report questionnaires were included that measured characteristics of childbirth memory (BirthMARQ), symptoms of psychopathology, and demographic information.

### Participants

Questionnaires were completed by 523 women. Inclusion criteria stipulated that women were 18 years or over, could read English fluently, and had given birth within the past year (*M* = 23.7 weeks, *SD* = 14.0, range 0–51 weeks). Participants’ ages ranged between 18 and 47 (*M* = 28.5 years, *SD* = 5.4).

### Materials

#### Birth Memories and Recall Questionnaire (BirthMARQ)

Measures used in studies of memory characteristics in other populations were reviewed to ensure we covered all potentially important domains of memory characteristics [12-15,21-27]. The initial item pool of 59 items included a range of memory characteristics important for emotional (including traumatic) memories, such as items about a physical/bodily emotional reaction during recall [28], narrative coherence and fragmentation [12], and the centrality of the event in a person’s identity/life story [26]. Such items are unusual in memory characteristics questionnaires but are potentially important for memories of an emotional experience like childbirth. In addition, items were added that are rarely included in previous questionnaires but may be important, including: whether memory images were continuous or fragmented; closure, i.e. a subjective state reflecting the degree to which a recalled experience feels resolved [25,29]; distancing, i.e. the extent to which one tries to psychologically distance oneself from the remembered experience [24]; and the frequency of voluntary and involuntary recall.

This set of 59 items was organised into 15 sections designed to measure: type of representation (words or images), sensory detail, spatial qualities, overall memory and vividness, coherence, memory for emotions during birth, emotions felt during recall, remembered thoughts, centrality of the memory in identity/life story, distancing, frequency of voluntary and involuntary recall, confidence in accuracy of recall, reliving, closure, and perspective of recall (first-person, third-person, or both). Items were measured on Likert scales (1–7). Wording of items was phrased to make it clear that questions were about memories of childbirth. If items were adapted from existing questionnaires approval was sought from the authors [14,26].

Face validity was assessed by administering the draft BirthMARQ to five women who had experienced birth. No items were removed as a result but changes were made to the wording of some questions to make them clearer.

#### Post-traumatic stress disorder

PTSD was measured using the Posttraumatic Stress Diagnostic Scale (PDS) [30], a self-report questionnaire that assesses history of traumatic events and PTSD symptoms in accordance with DSM-IV criteria. The PDS was adapted so women reported symptoms in relation to childbirth. This measure allowed us to determine whether participants met diagnostic criteria for PTSD. These were: women experienced threat to their own/their baby’s life during childbirth or a threat to their physical integrity; responses of fear, helplessness or horror; and reported re-experiencing, avoidance and hyper-arousal symptoms; symptom duration greater than one month; and experience of significant distress [31]. As a diagnostic tool, the PDS has well-established reliability, specificity and sensitivity [30] so was used as a measure of probable PTSD.

#### Depression

Depression was measured using the Edinburgh Postnatal Depression Scale, a measure with high reliability, sensitivity, and specificity, widely used to assess postnatal depression. Scores range from 0–30 and a score >13 was used to signify probable depression [32].

### Procedure

Women were recruited via the internet to enable access to a large, diverse sample compared to traditional recruitment methods [33] and to provide access to a special population (women who had given birth in the past 12 months). The study was advertised on baby-related websites (e.g. http://www.babycentre.co.uk, http://www.netmums.co.uk). Eligibility criteria were clearly stipulated, all responses were anonymous and consent was obtained. The BirthMARQ was administered before measures of mental health to avoid a possible cuing effect. The study received ethical approval from the University of Sussex Research Governance Committee.

### Analysis

Principal components analysis (PCA) was selected as the method of component extraction for the BirthMARQ in order to reduce the initial pool of items to a smaller, more manageable set that explained the greatest proportion of unique and shared variance. Cronbach’s alpha was used to establish the level of internal consistency for each component. Spearman rank correlations were used to examine associations between components and between time since giving birth and the six components. To evaluate validity of the questionnaire quasi-independent variables were created for PTSD (none, probable) and depression (none, probable). Mann–Whitney tests were used to test whether component scores differed between women with and without probable depression or PTSD.

## Results

Sample characteristics are shown in Table 1. Most participants identified themselves as white/Caucasian, and married or cohabiting. Over 50% were educated to degree-level and employed in professional or managerial/technical roles.

**Table 1:** Sample demographic characteristics

		***N***	**%**
Marital status	Married	371	70.90
	Cohabiting	127	24.30
	In a relationship but not cohabiting	7	1.30
	Divorced	2	0.40
	Single	16	3.00
Ethnicity	White	495	94.60
	Multi-ethnic	20	3.50
	Asian or Asian British	3	0.60
	Black or Black British	3	0.60
	Other	2	0.40
Education level	Post-graduate degree or equivalent^a^	125	23.90
	First degree or other higher education^b^	157	30.00
	A levels, NVQ level 3, or equivalent^c^	93	17.80
	GSCE/O Level, NVQ Level 2, or equivalent^d^	66	12.60
	Trade apprenticeships and other	18	13.40
	No qualifications	64	12.20
Occupation^e^	Professional	58	11.10
	Management or technical	211	40.30
	Skilled (non-manual)	124	23.70
	Skilled (manual)	28	5.40
	Partly skilled	33	6.30
	Other	62	11.90
No. of children^f^	0	1	0.20
	1	338	64.60
	2	128	24.50
	3	41	7.80
	4	11	2.10
	5	3	0.60
	6	1	0.20

^a^Includes Masters, Doctoral, and Profession (MD, JD) degrees.

^b^Includes 4-year college degrees (BA, BSc).

^c^Includes 2-year college degrees (Associates).

^d^Includes high school diploma or GED.

^e^*N* = 516 because 7 participants were in the armed forces or occupation was not adequately described.

^f^Includes data for a participant whose most recent birth experience resulted in stillbirth.

### BirthMARQ analysis

Twenty-five items with non-normal distributions (skewness >1) were removed [34]. The initial PCA (varimax rotation, no factors specified) did not converge but a review of the components with eigenvalues >1 [35] and the scree plot [36] suggested the existence of six components. Three subsequent PCAs identified 11 items with communalities < .40 to be removed. The final PCA (see Table 2) resulted in 23 items loading on six components that accounted for 64% of the variance. Diagnostic statistics were confirmed against published criteria [37].

**Table 2:** Component loadings for items on the Memories for Birth Questionnaire (BirthMARQ)

**BirthMARQ components (% variance)**	**Component loading**	***h***^***2***^	**Cronbach's alpha**^**a**^
**Emotional Memory** (12.94)			0.81
My emotions at the time were extremely positive*	0.85	0.74	
My emotions at the time were extremely negative	0.81	0.68	
I experienced mixed positive and negative emotions at the time	0.72	0.54	
While recalling the birth now, my emotions are extremely positive*	0.69	0.58	
While recalling the birth now, I am experiencing mixed positive and negative emotions	0.66	0.47	
**Reliving** (10.98)			0.80
While remembering the birth now, I relive visual impressions I had during the birth	0.80	0.68	
While remembering the birth now, I relive the bodily sensations I had during the birth	0.74	0.63	
While remembering the birth now, I feel as though I am reliving it and it is happening now, not in the past	0.73	0.63	
While remembering the birth now, I relive the sound(s) I heard during the birth	0.71	0.68	
**Centrality of memory** (10.83)			0.80
The experience of birth has coloured the way I think and feel about other experiences	0.82	0.72	
The experience of birth has become central to the way I understand myself and the world	0.77	0.68	
The experience of birth was a turning point in my life	0.74	0.65	
I often think about the effects the experience of birth will have on my future	0.59	0.58	
**Sensory Memory** (10.47)			0.74
As I recall the birth, I can remember smells	0.82	0.71	
As I recall the birth, I can remember tastes	0.77	0.63	
As I recall the birth, I can remember sounds	0.65	0.51	
As I recall the birth, I can remember touch	0.61	0.47	
**Recall** (9.88)			0.84
My memory for the birth (or parts of the memory) comes to me 'out of the blue' without me trying to think about it	0.84	0.78	
Things that happen now can unexpectedly bring up memories of the birth (or parts of memories)	0.77	0.74	
*Since the birth, I have thought about it*^b^	*0.74*	0.64	
**Coherence** (9.00)			0.84
My memory for the birth comes to me as a logical, coherent series of events with no major gaps	0.87	0.81	
My memory for the birth is fragmented, i.e. it comes in bits and pieces with bits missing*	0.85	0.75	
*The images in my memory for the birth come to me like a continuous film or video recording*^b^	*0.60*	0.46	

*Indicates the item should be reverse scored.

^a^Cronbach's alpha values presented here are for only those items included in the final subscale score calculations.

^b^Although this item loaded strongly with the others in this component, subsequent Cronbach's alpha analyses indicated the subscale internal consistency would be improved if the item were deleted. Subscale scores were calculated based on the remaining items.

The rotated component matrix was used to interpret the components. *Emotional Memory* (variance: 12.94%) included five items relating to the valence of emotions, both at the time of the birth and during recall. Higher scores on this scale indicate more negative emotion and/or more mixed emotion. *Reliving* (variance: 10.98%), a four item component, captured reliving of the experience through visual impressions, sound and bodily sensations, and how much it was re-experienced as if in the present. Increasing values indicate more reliving. *Centrality of Memory* (variance: 10.83%) included four items measuring the extent to which the memory of the birth experience was integrated as a central experience in the mother’s self-identity and life story. Higher scores indicate that the birth memory is more central to the mother’s identity. *Sensory Memory* (variance: 10.47%), a four-item component, assessed how well participants remember details of smells, tastes, sounds and touch at the time of the birth, with high scores indicating increased sensory memory. *Involuntary Recall* (variance: 9.88%) included three items assessing the frequency of involuntary, voluntary, and cued recall of the birth. One item assessing frequency of voluntary recall was later removed to improve internal consistency. Higher scores indicate greater frequency. *Coherence* (variance: 9.00%) consisted of three items that assessed the degree to which the birth memory was coherent, un-fragmented and like a continuous film. Again, one item assessing image continuity was later removed to improve internal consistency. Higher scores indicate more coherent memories.

#### Internal consistency

Two items were eliminated from the final set of items (see Additional file 1) and subscale score calculations because Cronbach’s alpha analyses indicated that this would improve values. One item was from *Involuntary Recall* (frequency of thinking about the birth, i.e. voluntary recall) and one from *Coherence* (memory images come to me like a continuous film). Internal consistency values for the final subscales are provided in Table 2. The mean of all alphas was 0.80. Usually alphas of 0.70 and above are considered acceptable for use with group-level comparisons [38]. According to this criterion, all of the subscales are suitable for use.

#### Correlations between components

Correlations between the six components are shown in Table 3. Many of the BirthMARQ components were significantly correlated but the effect sizes for most of the coefficients were low. The strongest relationships, with medium effect sizes (*r* = .31 to .47), were positive associations between *Reliving* and *Centrality of Memory*, *Sensory Memory* and *Involuntary Recall*; *Centrality of Memory* and *Involuntary Recall*; and *Sensory Memory* and *Coherence*. The length of time since giving birth was not associated with scores on any of the six subscales (all *r* < .10 and *p* > .05).

**Table 3:** Correlation matrix between BirthMARQ components and time since birth

**Component**	**1**	**2**	**3**	**4**	**5**	**6**
1. Emotional memory						
2. Reliving	0.09*					
3. Centrality of memory	0.00	0.40***				
4. Sensory memory	−0.05	0.38***	0.26***			
5. Involuntary recall	0.21***	0.37***	0.47***	0.23***		
6. Coherence	−0.20***	0.07	0.03	0.31***	−0.03	
7. Time since birth (weeks)	−0.01	−0.08	−0.05	−0.02	0.00	−0.04

*p < .05, ***p < .001.

### BirthMARQ validity

Validity of the BirthMARQ was examined by determining whether component scores differed between women with and without probable depression or PTSD. In the current sample, 21% of women met the cut-off for probable depression (*n* = 111) and 4% met the criteria for probable PTSD (*n* = 21). The majority of women with PTSD also had probable depression (*n* = 19). Table 4 provides descriptive and inferential statistics by depression and PTSD classifications for all BirthMARQ components.

**Table 4:** Median BirthMARQ component scores by depression and post-traumatic stress disorder

		**Depression**			**Post-traumatic stress disorder**		
	**Overall**	**None**	**Probable**		**None**	**Probable**	
	**(*N*** **= 523)**	**(*N*** **= 412)**	**(*N*** **= 111)**		**(*N*** **= 502)**	**(*N*** **= 21)**	
Emotional memory	3.20	2.60	4.40	***	3.00	5.00	***
Reliving	3.50	3.25	4.00	***	3.50	4.00	
Centrality of memory	5.00	4.75	5.25	*	4.75	5.75	*
Sensory memory	3.75	3.75	3.75		3.75	2.75	*
Involuntary recall	4.50	4.50	5.50	***	4.50	6.00	***
Coherence	5.50	5.50	5.50		5.50	3.00	***

*p < .05, ***p < .00. NB. Range of scores = 1 to 7

Results showed differences between mothers with and without probable depression or PTSD. Women with probable depression reported more *Emotional Memory, Reliving* and *Involuntary Recall *(all *p* < .001) and more *Centrality of Memory* (*p* < .05) compared to women without. There was no difference in ratings of *Sensory Memory* or *Coherence*. Women with probable PTSD rated their memories higher for *Emotional Memory* (*p* < .001), *Involuntary Recall* (*p* < .001) and *Centrality of Memory* (*p* < .05), and lower for *Coherence* (*p* = .001) and *Sensory Memory* (*p* = .03) than women without. There was no significant difference in *Reliving* scores.

### Characteristics of birth memories

The overall median ratings for each BirthMARQ component across all mothers shown in Table 4 reveals that birth memories were rated as highly coherent, as central to the mothers’ identities, with highly frequent involuntary recall. They were rated as close to the middle of the range for sensory impressions, reliving, and the degree to which the emotions at the time and during recall were negative or mixed.

## Discussion

This study developed and evaluated a self-report measure of the phenomenological characteristics of memories of childbirth which may be important in postnatal mood and psychopathology. Principal components analysis identified six components that accounted for 64% of the variance, and the mean alpha scores indicated good reliability. Birth memories were rated as highly coherent and were experienced as if the original experience was relived. These memory qualities are characteristic of episodic memories for specific events such as childbirth [22]. Birth memories were also rated as central to the mother’s identity, as might be expected for such a salient life event. Consistent with previous findings [1] mothers also rated the emotions associated with their memories of birth as negative or mixed.

Analyses of differences in memory characteristics between women with and without probable psychological symptoms of depression and PTSD suggest the scale is valid. With regard to depression, women who exhibited symptoms of depression reported more intense mixed and negative emotions associated with their childbirth memories, more frequent involuntary recall, more reliving, and memories that were more central to their identity. There was no difference between depressed and non-depressed mothers in ratings of sensory memory or coherence. These findings are largely consistent with existing research evidence. Previous studies have shown the same findings with regard to emotional memory, centrality of memory, sensory memory and coherence: compared with those who are not depressed, depressed individuals report more intense and more negative memories [17-19], memories that are more central to their life stories [19] but no difference in sensory detail or coherence [17,18]. Although some previous studies have found no association between depression and reliving or involuntary recall [19,20], our findings are consistent with previous studies that have found more reliving [39] and greater perceived uncontrollability of recall [17] in those who are depressed.

Mothers with probable PTSD reported more emotional memory, more involuntary recall, memories more central to their identities, less sensory memory and less coherence. The degree to which memories were relived did not differ between women with and without probable PTSD. For emotional memory, involuntary recall, centrality and coherence, these results are consistent with previous research. Berntsen *et al.*[15] found more reliving of emotion, more involuntary recall, and memories that were more central to identity in trauma memories of students with a PTSD profile compared with those without. High involuntary recall ratings from mothers with probable PTSD are also consistent with Ehlers and Clark’s [40] model of PTSD in which trauma memories are more susceptible to automatic cueing. The centrality of birth memories for mothers with probable PTSD supports Berntsen *et al*.’s [15] argument that in PTSD trauma memories are well-integrated into the life story as a landmark. In our study, mothers with probable PTSD rated their memories of childbirth as less complete and coherent. While previous evidence is inconsistent with regard to coherence [12,15,41], our results support Brewin’s [16] conclusion following a review of the evidence that, in clinical populations, trauma memories are more fragmented, disorganised and incomplete for memories recalled voluntarily. They are also consistent with the models of PTSD proposed by Brewin, Dalgleish and Joseph [42] and Ehlers and Clark [40] and with the results of the two previous studies that have examined the coherence of memories for childbirth in women with PTSD symptoms [7,8].

Contrary to expectations, mothers with probable PTSD did not report greater reliving than those without, and they reported *fewer* sensory impressions. PTSD is usually associated with vivid reliving and sensory impressions [12,15,16]. One possible reason for this difference is that in this study childbirth memories were recalled voluntarily, while vivid reliving and sensory memory are usually associated with involuntary, intrusive memories of trauma. Taken together, the differences between memory characteristics for women categorised by depression and PTSD symptoms are mostly consistent with theoretical accounts and existing literature, thus supporting the validity of the BirthMARQ. However, the inconsistencies require further research and exploration.

Similarly, there are limitations to the current study that future research should address. Firstly, concurrent validity was not measured against other memory characteristic questionnaires. Although the BirthMARQ is unique in the sense that it is the only memory questionnaire developed specifically for measuring childbirth memory characteristics, comparison with general memory characteristic questionnaires would nevertheless be informative. Secondly, psychological symptoms were measured using self-report questionnaires. Scores on these measures of depression and PTSD can only indicate probable psychological symptoms. Therefore, repeating this analysis using a clinical sample of women diagnosed with postnatal depression or postnatal PTSD would be constructive. Future research could also examine the relationship between childbirth memory characteristics and postnatal anxiety disorders other than PTSD. Thirdly, as yet, no test-retest reliability has been carried out so it is important to further test the reliability of the tool. Fourthly, childbirth often involves the use of analgesia. The effect of analgesia on birth memories is unclear but some studies have shown it can cause varying degrees of amnesia [43-45] and Briddon *et al*. [7] suggest that analgesia with greater consciousness-affecting (rather than pain-affecting) properties may be associated with more disorganised memories of birth. Thus the use of analgesia during labour might result in some inaccuracy or gaps when recalling the birth. Further research is needed on the reported amnesic effect of analgesia in the context of birth memories and the BirthMARQ questionnaire would be a useful tool for this.

## Conclusions

The BirthMARQ is the first questionnaire specifically designed for measuring characteristics of memories of childbirth that may be important in postnatal mood and psychopathology. It was developed from a wide range of memory characteristics that previous research suggests are important and includes items characterising emotional and traumatic memories. The results predominantly support the BirthMARQ’s internal consistency and validity. While further testing of its reliability and validity is needed, it is a measure capable of becoming a valuable tool for examining memory characteristics for the important event of childbirth. This includes the association between memory characteristics and psychological and physical outcomes, such as obstetric events, postnatal mood, social disclosure, infant temperament, and the mother-baby bond. For example, if differences in memory characteristics between subgroups of women were central to the research question, such as those who experience depression or PTSD following birth, then the subscales shown to discriminate these groups would be of most interest. There are also potential clinical applications of the BirthMARQ. For instance, the emotional memory subscale could be used to screen for emotion dysregulation in women who have experienced a traumatic birth. Emotion dysregulation has been associated with PTSD and screening could facilitate appropriate emotion regulation interventions [46]. Finally, use of this questionnaire need not be restricted to measuring women’s memory for childbirth but could also be applied to examine men’s memories for the birth of their children, subject to validation.

## Abbreviations

BirthMARQ: The Birth Memories and Recall Questionnaire; MEQ: Memory Experiences Questionnaire; PCA: Principal components analysis; PDS: Posttraumatic Stress Diagnostic Scale; PTSD: Post-traumatic stress disorder.

## Competing interests

The authors declare that they have no competing interests.

## Authors’ contributions

All authors contributed to drafting the manuscript and read and approved the final manuscript. SF contributed to the conception and design of the study, developing the questionnaire, collecting the data and doing preliminary analyses. RC contributed to the conception of the study, developing the questionnaire and oversaw drafting of the manuscript. SW carried out the statistical analyses. SA contributed to conception and design of the study and analyses.

## Pre-publication history

The pre-publication history for this paper can be accessed here:

http://www.biomedcentral.com/1471-2393/14/211/prepub

## Supplementary Material

Additional file 1 Birth Memories and Recall Questionnaire (BirthMARQ). Click here for file
