# Supplementary material for: The Birth Memories and Recall Questionnaire (BirthMARQ): development and evaluation
Source: BMC Pregnancy Childbirth. 2014 Jun 20;14:211. doi: 10.1186/1471-2393-14-211 (PMC4229988; doi:10.1186/1471-2393-14-211)
Supplement: Additional file 1 — Birth Memories and Recall Questionnaire (BirthMARQ). [file 1471-2393-14-211-S1.docx]

Additional file 1

Birth memories and recall questionnaire (BirthMARQ)

This questionnaire asks about what your memories of the birth of your most recent child are like, and how you feel when you remember the birth now. If you had a caesarean under general anesthetic, please answer the questions as best you can for your memory of the experience immediately before and after.

|  | Emotional Memory | Strongly Disagree |  |  |  |  |  | Strongly Agree |
| --- | --- | --- | --- | --- | --- | --- | --- | --- |
| 1* | My emotions at the time were extremely positive | 1 | 2 | 3 | 4 | 5 | 6 | 7 |
| 2 | My emotions at the time were extremely negative | 1 | 2 | 3 | 4 | 5 | 6 | 7 |
| 3 | I experienced mixed positive and negative emotions at the time | 1 | 2 | 3 | 4 | 5 | 6 | 7 |
| 4* | While recalling the birth now, my emotions are extremely positive | 1 | 2 | 3 | 4 | 5 | 6 | 7 |
| 5 | While recalling the birth now, I am experiencing mixed positive and negative emotions | 1 | 2 | 3 | 4 | 5 | 6 | 7 |
|  | Reliving | Not At All |  |  |  |  |  | Very Much So |
| 6 | While remembering the birth now, I relive visual impressions I had during the birth | 1 | 2 | 3 | 4 | 5 | 6 | 7 |
| 7 | While remembering the birth now, I relive the bodily sensations I had during the birth | 1 | 2 | 3 | 4 | 5 | 6 | 7 |
| 8 | While remembering the birth now, I feel as though I am reliving it and it is happening now, not in the past | 1 | 2 | 3 | 4 | 5 | 6 | 7 |
| 9 | While remembering the birth now, I relive the sound(s) I heard during the birth | 1 | 2 | 3 | 4 | 5 | 6 | 7 |
|  | Centrality of memory | Strongly Disagree |  |  |  |  |  | Strongly Agree |
| 10 | The experience of birth has coloured the way I think and feel about other experiences | 1 | 2 | 3 | 4 | 5 | 6 | 7 |
| 11 | The experience of birth has become central to the way I understand myself and the world | 1 | 2 | 3 | 4 | 5 | 6 | 7 |
| 12 | The experience of birth was a turning point in my life | 1 | 2 | 3 | 4 | 5 | 6 | 7 |
| 13 | I often think about the effects the experience of birth will have on my future | 1 | 2 | 3 | 4 | 5 | 6 | 7 |
|  | Sensory Memory | None At All |  |  |  |  |  | A Lot |
| 14 | As I recall the birth I can remember smells | 1 | 2 | 3 | 4 | 5 | 6 | 7 |
| 15 | As I recall the birth, I can remember tastes | 1 | 2 | 3 | 4 | 5 | 6 | 7 |
| 16 | As I recall the birth, I can remember sounds | 1 | 2 | 3 | 4 | 5 | 6 | 7 |
| 17 | As I recall the birth, I can remember touch | 1 | 2 | 3 | 4 | 5 | 6 | 7 |
|  | Recall | Never |  |  |  |  |  | All The Time |
| 18 | My memory for the birth (or parts of the memory) comes to me 'out of the blue' without me trying to think about it | 1 | 2 | 3 | 4 | 5 | 6 | 7 |
| 19 | Things that happen now can unexpectedly bring up memories of the birth (or parts of memories) | 1 | 2 | 3 | 4 | 5 | 6 | 7 |
|  | Coherence | Strongly Disagree |  |  |  |  |  | Strongly Agree |
| 20 | My memory for the birth comes to me as a logical, coherent series of events with no major gaps | 1 | 2 | 3 | 4 | 5 | 6 | 7 |
| 21* | My memory for the birth is fragmented, i.e. it comes in bits and pieces with bits missing | 1 | 2 | 3 | 4 | 5 | 6 | 7 |

* Indicates the item should be reverse scored.
